# Shared Components of Worldwide Successful Sexuality Education Interventions for Adolescents: A Systematic Review of Randomized Trials

**DOI:** 10.3390/ijerph20054170

**Published:** 2023-02-25

**Authors:** Betzabé Torres-Cortés, Loreto Leiva, Katia Canenguez, Marcia Olhaberry, Emmanuel Méndez

**Affiliations:** 1 Department of Psychology, Faculty of Social Sciences, Pontificia Universidad Católica de Chile, Av. Vicuña Mackenna 4860, Macul 7820436, Chile; 2 Department of Psychology, Faculty of Social Sciences, Universidad de Chile, Avenida Capitán Ignacio Carrera Pinto 1045, Ñuñoa 7800284, Chile; 3 Millennium Institute for Research on Depression and Personality (MIDAP), Av. Vicuña Mackenna 4860, Macul 7820436, Chile; 4 Department of Psychiatry, Massachusetts General Hospital, Yawkey 6A, 55 Fruit Street, Boston, MA 02114, USA; 5 Department of Psychiatry, Harvard Medical School, 25 Shattuck St, Boston, MA 02115, USA; 6 Department of Psychiatry, Faculty of Medicine, Universidad de Chile, Gran Av. José Miguel Carrera 3100, San Miguel 8900085, Chile

**Keywords:** sexuality education interventions, adolescents, systematic review, randomized controlled trials, shared components

## Abstract

A crucial aspect of human development is sexuality which has implications for health, particularly in adolescence, since unfavorable sexual experiences may result in physical and mental problems. Sexuality education interventions (SEI) are one of the most used actions to promote sexual health in adolescents. Nevertheless, there is variability across their components; therefore, key elements for an effective SEI targeted at adolescents (A-SEI) are not well known. Based on this background, this study aims to identify the shared components of successful A-SEI through a systematic review of randomized controlled trials (RCT). This study followed the preferred reporting items for systematic reviews and meta-analyses statement. A search was conducted in CINAHL, PsycInfo, PubMed, and Web of Science between November and December 2021. A total of 21 studies passed the inclusion test after the review of 8318 reports. A total of 18 A-SEIs were identified in these studies. The components analyzed were the intervention’s approach, dose, type of intervention, theoretical framework, facilitators’ training, and intervention methodology. The results established that components that should be present in the design of an effective A-SEI are behavior change theoretical models, the use of participatory methodology, be targeted at mixed-sex groups, facilitators’ training, and at least ten hours of weekly intervention.

## 1. Introduction

Sexuality is a central dimension of human development [1], mainly throughout adolescence, with implications for physical and mental health [2,3]. Indeed, certain negative sexual experiences could lead to sexually transmitted diseases (STI) such as gonorrhea and syphilis, human immunodeficiency virus (HIV), as well as nonintended pregnancy, maternal complications, intimate partner violence, depression, suicidality, and anxiety [4,5,6,7,8,9,10]. 

One of the key strategies to address these challenges are sexuality education interventions (SEI), which represent one of the most used preventive actions [2]. These interventions can be delivered in person, remotely (through a computer or text messages), or in a mixed way. 

Some of the benefits of adolescent sexuality education include gaining a greater understanding of expected body changes, an important educational component that helps youth emotionally prepare for these changes [11,12]. In addition, SEI is associated with the delay of sexual onset, given that informed sexual decision making promotes a healthier sex life and an overall reduction in adverse sexual risk behaviors [13,14,15,16,17]. However, despite these favorable outcomes, there is great variability in the SEI curriculum with varied components. This makes it challenging to understand which are the key elements that lead to favorable outcomes [18].

### 1.1. Sexuality Education Intervention Approaches

#### 1.1.1. Abstinence Interventions

SEI interventions are varied as the approaches used in their designs are diverse. For example, the abstinence approach promotes the delay of sexual intercourse until marriage [19]. Currently, there are two types of abstinence intervention. The first is abstinence-only, whose unique objective is to promote abstinence as well as psychological and health benefits [20]. It is an approach frequently used in communities where, due to religious or cultural beliefs, sexual activities take place after marriage [21]. The second, abstinence-plus, promotes abstinence as well as sexual healthcare methods such as appropriate condom use and contraceptive use [22] which could reduce HIV risk [23].

Although some evidence has shown that abstinence-plus SEI could have favorable effects such as reducing HIV risk [23] and increasing levels of knowledge, self-efficacy, and positive attitudes towards abstinence [24], meta-analyses and systematic reviews [20,23], as well as other studies [25,26], have demonstrated that, in general, abstinence SEI is not sufficiently effective due to its limited capacity to delay sexual debut and prevent pregnancy. In addition, some studies reveal that they do not meet the needs of sexually active adolescents, reinforce gender stereotypes [27], and promote stigmas and mental health problems in lesbian, gay, bisexual, and transgender (LGTB) adolescents [28]. 

Of note, abstinence-plus interventions have been referred to as “comprehensive”’ [23,29]. However, others would not recognize this intervention as a “comprehensive” SEI intervention [22]. In this research, the comprehensive approach will be postulated as different from the abstinence-plus approach.

#### 1.1.2. Comprehensive Interventions

The comprehensive approach understands sexuality as multidimensional so covers biological, psychosocial, and value-based aspects [30]. The concept “comprehensive” is used as a flexible way to describe a variety of programs [31], which have in common a teaching process that emphasizes the advantages of abstinence and provides information on sexual self-care methods [19]. Comprehensive SEI’s primary focus is not just on sexual behavior change but promoting safe sexuality and satisfying sexual needs [32] through culturally competent interventions [33]. In addition, it has a human rights and gender equity approach [34,35] and includes sexuality-related aspects of mental health to promote well-being [2]. 

There is consistent evidence on the comprehensive intervention’s effectiveness [36] as meta-analysis [20] and other studies [37,38] noted that it is the most effective strategy for reducing pregnancy and STDs. Moreover, there is evidence of their favorable impact on mental health since these interventions contribute to increased self-esteem and self-efficacy; decreased depressive symptomatology; improved sense of self-control, self-confidence, and self-image; the promoting of general psychological adjustment; increased well-being and self-esteem in female and LGBT adolescents; and decreased suicidality in LGTB adolescents [2,38,39,40]. 

Nonetheless, despite the available evidence, comprehensive interventions are less studied than abstinence or risk approaches [38], and as a result, their effectiveness and implementation knowledge are limited [41]. Therefore, some research indicates that comprehensive approaches do not always lead to gender-transformative outcomes [42], and they have shown a deficit in consistently addressing mental health [43]. 

Some authors point out that abstinence and comprehensive approaches are the predominant perspectives [29] but others note a third approach aimed to promote safe sexual practices by emphasizing the risks of unsafe ones, called risk-oriented interventions [21]. 

#### 1.1.3. Risk-Oriented Interventions

The risk-oriented approach aims at biological aspects of sexuality with topics such as pregnancy, STIs, and contraception based on the concern about behavioral and external factors that can impact health; thus, it usually does not include topics such as rights, pleasure, and gender equity [44,45,46].

A review by Sales [47] notes that risk-oriented interventions effectively reduce the frequency of unprotected sexual activity and the number of sexual partners and delay the onset of sexual intercourse. However, the results of this kind of SEI are primarily associated with knowledge, which does not necessarily imply significant behavioral changes [48]. On the other hand, they do not enable empowerment and deliver limited information about pleasure, excluding relevant topics for adolescents, such as those related to gender [45,49].

Each of these approaches takes a different perspective to educating youth about sexuality. As we develop evidence-based interventions, we must identify the key elements that lead to change and favorable outcomes. 

### 1.2. Additional Components of Sexuality Education Interventions for Adolescents

In addition to the intervention approach, there are other sensitive elements involved in the development of health interventions. These components are related to fidelity, e.g., the training of the facilitators; delivery, e.g., the frequency of contact; and engagement elements, e.g., rewards for participation [18].

#### 1.2.1. Theoretical Framework

Theories are relevant in evidence-based health promotion interventions because they address change factors and allow for better decisions in their design [50].

The most used theories among interventions oriented to sexual behavior change are the social cognitive theory, the theory of reasoned action, the health belief model, health behavior theory, and the information, motivation, and behavioral skills model [22,51,52]. However, it has also been suggested that is necessary to include theories that address relational, cultural, and socioeconomic factors influencing sexual behavior [53]. 

#### 1.2.2. Type of Intervention

Evidence shows that more effective interventions are those for groups [38]. However, certain SEIs delivered individually have achieved favorable results [54,55]. On the other hand, mixed-sex group interventions, in comparison with single-sex ones [38], have demonstrated higher effectiveness. Although, there are also some studies that claim that single-sex interventions work in specific situations that focus on cultural or religious sensitivities, those with high-risk populations, or when there are different degrees of knowledge in men and women [56,57].

#### 1.2.3. Dose

The dose refers to the amount of exposure to an intervention [58] and is a component with high variability in SEI targeted at adolescents (A-SEI). The number of sessions and their duration are changeable as interventions with favorable results may have 1 [59], 6 [60], 10 [37,61], or up to 14 sessions [62]. Likewise, the duration also fluctuates since sessions can last 45 min [62,63], 50–60 min [64], or 4 h [65]. Therefore, even when the dose is considered, the moderator of SEI effects is not clear [58], and there is no certainty about the dose–effectiveness relation in this type of interventions [66]. 

#### 1.2.4. Intervention Methodology

There are two types of strategies for delivering the intervention: the expository and the participatory methodology. The expository methods use discourse and the explanation of content is led by the facilitator, including lectures, discussions, and oral reports [67,68,69,70,71,72,73,74,75,76,77,78,79]. 

Certain authors point out that expository activities allow the dissemination of knowledge on human sexuality, especially in large groups, and focus on controversial topics [67]. Nevertheless, some state that these techniques establish a vertical teacher–student relationship where students must assimilate content; in contrast, sex education requires a climate of dialogue and trust [70]. 

On the other hand, the participatory method implies a horizontal relationship, promotes knowledge production, and allows participants to be reflective individuals [71]. Some studies show that these methods are the most effective for teaching about sexuality, as they can engage adolescents’ attention, lead to behavior change, and contribute to preparing them for life by favoring their health in a complex and changing world [72,73]. 

#### 1.2.5. Facilitator’s Training

This is a critical element considered to be an implementation facilitator [74]. How-ever, some authors point out that only some SEIs include training; therefore, facilitators without preparation consider themselves not to have suitable skills or feel uncomfortable teaching about sexuality [75]. 

To summarize, there is a lack of agreement on the components that SEIs targeted at adolescents (A-SEI) must have as a “gold standard,” which may lead to a differential impact of the intervention [76,77]. In addition, there are no recent high-quality studies on their specific components [41], as most of the research focuses on pregnancy and STD indicators [39]. 

As a result, and despite being an important topic, little is known about what the key effective SEI components are. In fact, intervention designers have limited knowledge of the components that an SEI requires to be effective. In summary, further studies are required to help clarify what the effective components of the intervention are, which can help guide the design or adaptation of future interventions [38,60,67]. Systematic reviews have addressed this gap in different areas of health promotion by evaluating the characteristics of successful interventions which contribute to the development of or improvement in programs, such as Brook [78], Murimi [79], Pinto [80], and Ramage [81]. By identifying key features and common characteristics of these interventions, recommendations can be provided to designers and implementers of such intervention. 

This study aims to identify the shared components of successful A-SEI interventions through a systematic review of randomized control trials (RCT) over the last ten years. Study outcomes will be identified to gain a better understanding of their impact on health promotion. Thus, this review will allow us to identify common characteristics of successful interventions, which will help develop guidelines for an improved A-SEI design. 

The protocol for the present study was not published prior to conducting the review; however, it is available in the Supplementary Materials (S1).

## 2. Materials and Methods

This study was conducted following the Preferred Reporting Items for Systematic Reviews and Meta-analyses (PRISMA) statement [82].

### 2.1. Eligibility Criteria

The study selection process involved the screening of titles, abstracts, and full texts by two researchers to check the eligibility criteria, independently. When they had doubts as to whether the article would meet the eligibility criteria, other researchers resolved the discrepancies. In all cases, all the authors of this study made the final eligibility decision.

The following inclusion criteria were applied for screening:

(1) Publication date: studies published from 2011 onwards.
(2) Language: written in English or Spanish.
(3) Intervention: Any combination of learning experiences aimed at developing a voluntary behavior leading to sexual health in adolescents [83]. The intervention had to be universal, preventive, targeted at adolescents (11 to 19 years old), and only include sex health-related topics (from the abstinence, risk-oriented, or comprehensive approach). This study included interventions that targeted strictly sex behavior (and not those that addressed exclusively related topics as partner violence or sexual abuse). Only in-person SEI interventions were included in this review because of the differences in emphasis and methodologies between in-person and remote health education interventions (e.g., physical activity attention to educational material; group interaction versus one-to-one accountability) [84,85,86]. For this review, we established two categories to define the type of intervention: (a) level of intervention (individual or group), and (b) sex of the participants (single-sex or mixed-sex groups).
(4) Study design: experimental design (randomized control trial (RCT))
(5) Intervention outcomes: intervention with at least one statistically significant positive outcome.

On the other hand, the following exclusion criteria were applied:

(1) Not an empirical design: the study was a systematic review, protocol, or meta-analysis.
(2) Not face-to-face intervention: the intervention was a remote (delivered completely or in part via the internet, a video, or a text message) or computer-based SEI.
(3) Other participants: the intervention included parents or caregivers as active participants or was targeted at individuals with high risk in sex health.

No restrictions were included related to the intervention setting.

### 2.2. Information Sources

Search was conducted in CINAHL, PsycInfo, and PubMed databases and the Web of Science (WoS) platform between November and December 2021. These sources were chosen due to the quality of their journals and because they contain studies specialized in psychological or health science. Search terms were obtained based on a previous A-SEI general literature review. These terms are included in Table 1. The filters and limits used for the search can be seen in Supplementary Materials (S2).

### 2.3. Data Collection

The inclusion criterion number one was included in the search in the databases and platform. In this way, the search only included studies from 2011 onwards. Then, two researchers independently conducted the next steps. First, the duplicated articles were eliminated with the Rayyan automatized tool [87]. Second, in the article’s title, inclusion criteria number two, three, and four and exclusion criteria number one and two were screened. Additionally, duplicate articles were manually revised. Third, in the cases where this information was not available in the title, the abstract was revised for inclusion criteria number three and four and exclusion criteria number one and two. Inclusion criterion number five and exclusion criterion number three were screened. The search of the titles and abstracts was conducted using the Rayyan systematic review software. Fourth, in the cases where the abstract did not provide enough information about the eligibility criteria, it was proceeded to revise in full text. Finally, the studies were evaluated with the Jadad scale [88]. The studies that scored three or more points in this last evaluation were coded.

For the codification (conducted by two researchers independently), a datasheet for the coding process was developed. The study variables included were: the author, the year of publication, the title, the country of implementation, the type of randomization, blinding, the existence of a control group, the reasons for withdrawal, and the sample characteristics (size, sex, gender, and age). In addition, the following intervention characteristics were coded: the name of the intervention, the country of implementation, the objective, the setting (communitarian, scholar, or clinical), the number and length of sessions, the type of facilitators, the facilitators’ training, theoretical foundations, topics, the type of intervention (individual or group; single-sex or mixed-sex groups), the type of activities, and the statistically significative outcomes. 

The other variables coded were: the reported language, the type of publication, the founding sources, the type of RCT, the reward for participation, the sample size (experimental and control group), the amount of data analyzed pre- and post-intervention, the instruments applied, and the dropout rate. 

In cases where the information could not be found in the selected study, electronic mail was sent to authors to request missing information. 

### 2.4. Risk of Bias and Assessment of Study Quality

To ensure the quality of the studies, high-quality indicators were included in the eligibility criteria: the experimental design and existence of a control group [89]. The Jadad was also utilized [88]. This is one of the most used scales for evaluating RCTs. It rates the study as weak (zero points) to good (five points) and can be used for different types of studies, populations, and health approaches [90]. This scale contains items directly associated with the reduction in bias and classifies the study as high quality when this goes from three to five points [88].

### 2.5. Synthesis Method

For this review, a structured approach was used, as the intervention components of each study were coded in a database on prespecified criteria (e.g., approach: (1) abstinence, (2) comprehensive, (3) risk-oriented). Through this method, those with similar codes were grouped into the same categories.

## 3. Results

### 3.1. Study Selection

A total of 9131 studies were found in all sources. After eliminating duplicates with the automatized tool (*n* = 813), there were 8318 remaining studies. Following this, the title was revised, and 7306 studies were eliminated because (i) the intervention was not a universal preventive A-SEI (*n* = 6793), e.g., the intervention covered other topics such as nutrition, drug abuse, infertility, and intimate partner violence; (ii) the article was a systematic review, protocol or meta-analysis (*n* = 100); (iii) the intervention included or was targeted at other participants (*n* = 340)—targeted populations such as adults, HIV-positive individuals, or people with psychiatric diagnoses; (iv) the intervention was remote or computer-based (*n* = 57), (v) the study did not have an experimental design (*n* = 1); and study duplicates (*n* = 15).

Then, the abstracts of 1012 studies were revised, and 649 studies were eliminated because (i) the intervention was not a universal preventive A-SEI (*n* = 237), e.g., the intervention included the prevention of alcohol use or drug abuse or sexual victimization; (ii) the article was a systematic review, protocol, or meta-analysis (*n* = 120); (iii) the study did not report quantitative measures (*n* = 146); (iv) the intervention included or was targeted at other participants (*n* = 106); the intervention targeted populations such as adults, young adults, or persons who inject drugs; and (v) the study did not have an experimental design (*n* = 40).

When the full texts of 363 studies were screened, 336 studies were eliminated because (i) the intervention was not a universal preventive A-SEI (*n* = 47), e.g., the intervention included the prevention of school attrition, substance use, and delinquency; (ii) the intervention was remote or computer-based (*n* = 36); (iii) the study did not report quantitative outcomes (*n* = 45) or the study did not have an experimental design (*n* = 27); (iv) the intervention included or was targeted at other participants (*n* = 120), e.g., homeless youth, young fathers, or hospitalized adolescents; (v) article was not written in English or Spanish (*n* = 2) (German and Italian); and (vi) the intervention did not have any statistically significant outcome (*n* = 57).

**Figure 1:**
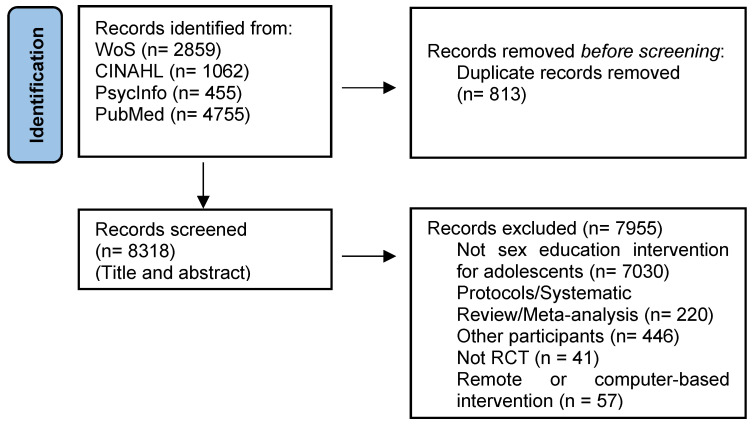

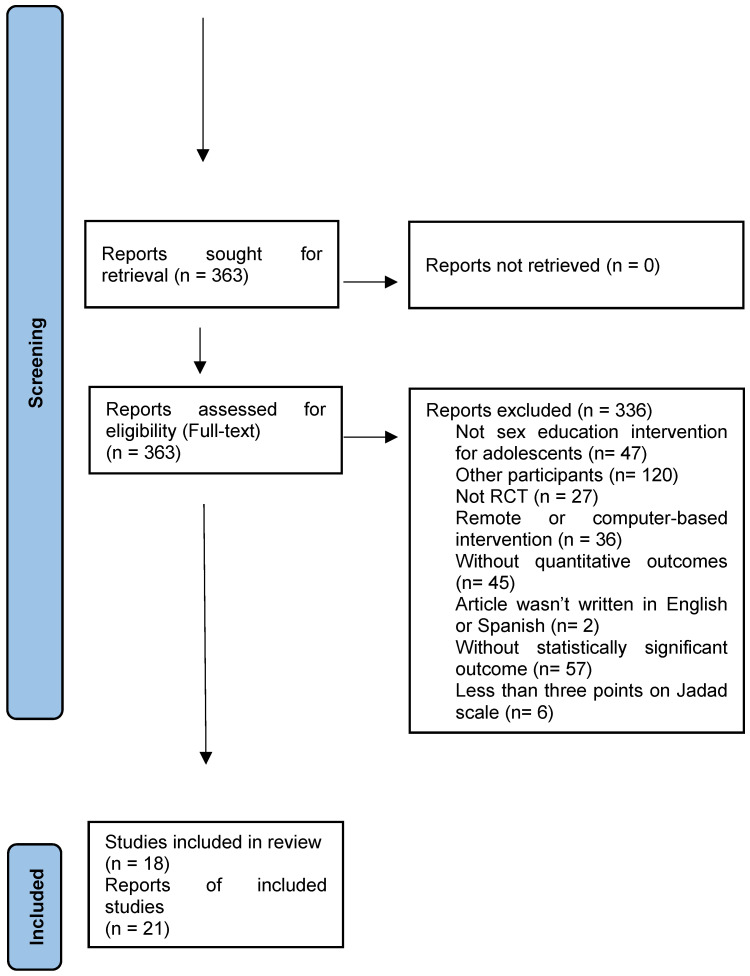
Preferred Reporting Items for Systematic Reviews and Meta-analyses (PRISMA) flow diagram study selection and inclusion process.

The final 27 studies remaining were evaluated with the Jadad scale [88]. Of these studies, 21 scored 3 or more points and 6 scored 2 points and were included in this review. See Table 2 for the characteristics of these studies and their Jadad scale score. The study selection and inclusion process can be seen in the PRISMA diagram (Figure 1).

### 3.2. General Characteristics of the Interventions

Within the 21 studies included, a total of 18 interventions were identified. In these studies, one intervention was reported in four studies included in this review [106,107,108,109]; another intervention was reported in two studies [39,98]; and one study reported two interventions [96].

The interventions were implemented in Africa, Europe, and North America, and most of the studies included in the review were conducted in educational settings, except for Bangi [95] and Barbee [96], who carried it out in community settings, and Bauman [96], whose research was conducted in a clinical setting. 

The intervention instructors were youth facilitators, school nurses, midwives, school teachers, professional actors, health educators, sexuality educators, research assistants, volunteer university students, and psychologists. The participants were between 11 and 19 years old. For more details about the studies included in this review, see Table 2.

### 3.3. Intervention Approach

Of the total number of interventions identified and included in this study (*n* = 18), 13 of them (72.22%) had a comprehensive approach. These interventions include topics related to the biological aspects of sexuality (e.g., sexual and reproductive anatomy, STI and pregnancy prevention, and condom use skills); psychosocial aspects (e.g., resilience, healthy relationships, relationships and emotions, assertive communication, self-efficacy, self-esteem, sexual violence, and HIV and stigma); values (e.g., abstinence and personal and community values); decision making (e.g., sexual risk behavior, refusal skills, coercion and consent, online safety, and goal setting); human rights (e.g., sexual rights); and gender (e.g., gender roles, norms and beliefs, sexual orientation, gender identity, power dynamics in relationships and in media messages, and gender power inequities). 

Four interventions (22.2%) used a risk reduction approach. These interventions addressed risk behaviors, contraception, condom use, HIV/STI risk reduction knowledge, sexual anatomy, reproductive systems, abstinence, communication about sex, emotion identification, self-efficacy, decision making, and negotiation about abstinence and condom use.

Finally, one intervention (6%) had an abstinence-plus approach. This intervention was oriented to sexual and prophylactic intentions and behavior, as well as attitudes, norms, motives, and knowledge about pregnancy and contraceptive methods. See Table 3 for the approach of every intervention.

### 3.4. Dose

There was great variability in the number of sessions and the total hours of exposure to the intervention. The number of intervention sessions went from 1 to 25: 44.44% between 11 and 16 sessions, 22.2% between 5 and 9 sessions, 17% between 21 and 25 sessions, and 17% of the interventions were composed of 1 session.

In terms of the total hours of intervention, the exposure went from 1 to 26 h: 50% between 10 and 19 h of exposure, 22.2% between 1 and 5 h, and 11.1% between 25 to 36 h. It was not possible to find this information for two of the programs [93,104]. 

With respect to frequency, of the 15 interventions with more than 1 session, 60% were delivered weekly, 13.33% daily, 7% monthly, and 20% were distributed differently (from 2 weeks to 4 months, across an average span of 53 days and 21 (or fewer) days). See Table 3 for the dose for each intervention.

### 3.5. Theoretical Frameworks

Most of the interventions included Ajzen’s theory of planned behavior [110] or theory of reasoned action [111,112], which is the TPB’s foundational framework, within their theoretical basis (61.1%). The second-most-used theory (39%) was Bandura’s social cognitive theory [113]. It is noteworthy that 39% of the interventions included two or more theoretical frameworks. The theories most used all together in one intervention were the theory of planned behavior and social cognitive/learning theory.

With regard to interventions that used the theory of planned behavior, they mainly focused on changing attitudes, perceived norms, and behavioral intentions about sex behavior risk [99]. The interventions based on the theory of reasoned action focused on how others would perceive the sex behavior [97]

Interventions that had Bandura’s social cognitive theory in their theoretical foundation considered that sex behavior was related to social influences, and motivation and personal skills were fundamental to change it [106]. 

It was not possible to find this information for one intervention [92]. See Table 4 for the theoretical framework for each intervention.

### 3.6. Type of Intervention 

All the interventions in this review were implemented with groups. Most of them (94.4%) were targeted at mixed-sex groups. Only one intervention [94] was delivered to a single-sex group (African American female adolescents).

### 3.7. Intervention Methodology

Although the A-SEIs included in this review used a variety of strategies, most of them can be classified within the participatory methodology. As a result, 94.4% of them used a participatory–interactive methodology. This included strategies such as icebreakers, games, demonstrations, brief lectures, worksheets, group discussions, critical thinking activities, role-plays, photo-novellas, stories, problem solving, exercises, music, and group activities (e.g., artistic expression). Only one intervention [94] used a different methodology, with discussion as the main technique. 

On the other hand, 33.3% of the interventions [92,94,95,96,105] included one or more videos in the sessions. See Table 3 for the intervention methodology for each intervention.

### 3.8. Facilitator’s Training

Most of the interventions (94.4%) gave training to their facilitators (see Table 2), between two days and two weeks in length, mainly on the intervention curricula and their core components, how to complete the registers, protocols to interact with adolescents, and how to answer sensitive questions.

The training was given through in-person sessions, online tutorial sessions, training and intervention manuals, demonstration videos or audios, the literature about session topics, and pilot training. In some training sessions, the facilitators modeled the intervention activities through role playing and received feedback. Therefore, in some cases, the facilitators received monitoring and assistance during the study.

However, it was not possible to find this information for one intervention [92].

### 3.9. Intervention Outcomes: Incidence on Health

In addition to the components of the intervention, we identified their main outcomes. The impact of these interventions reached different aspects of health.

First, the interventions produced psychosocial outcomes. This resulted in better attitudes toward condom use, partner communication about STIs, relationship rights, HIV testing, and people living with HIV. Additionally, there was higher perception of the severity of teenage pregnancy, barriers to adolescent pregnancy prevention, sexual and STI risk, the benefits of delaying pregnancy, and peers’ consistent condom use. In addition, participants achieved greater self-efficacy and lower rates of intimate partner violence. Finally, greater intention for communication about sex, pregnancy, relationships, and STIs with partners or parents was observed, as well as an increase in the intention to abstain from sex and to use condoms.

Second, the interventions increased knowledge on sexual and reproductive health, sexual health services, and condom/contraceptive use. Furthermore, the participants obtained a greater amount of information about susceptibility to adolescent pregnancy, sexual risk taking, pregnancy, and STI transmission and protection.

Some outcomes were associated with preventive sex behavior, such as: age delay of first intercourse; abstinence; a decrease in the incidence of sexual initiation and recent sex; reduced pregnancy risk; less risky behavior (e.g., unprotected sex); fewer sexual partners; and the use of birth control, condoms, and sexual health services.

See Table 5 for detailed intervention outcomes.

## 4. Discussion

This study aimed to identify the shared components of successful A-SEIs through a systematic review of RCTs conducted over the last ten years. A total of 21 articles met the inclusion criteria and obtained 3 or more points in the Jadad scale. In these articles, eighteen interventions were evaluated.

In terms of shared components, this review concludes that a comprehensive approach was present in the design of most A-SEI (78%), being consistent with the evolution of these interventions from a risk approach to a more holistic and structural one [127]. Furthermore, the relevance of this approach to fulfill the goals of SEI and to obtain positive outcomes also have been reported by similar studies [128,129]. Finally, it is important to mention that the literature suggests that sex education should engage young people in building their sexual and reproductive future [66], which is one of the focuses of comprehensive interventions that seek the promotion of safe and satisfying sexuality [32].

With regard to theoretical basis, 61.1% of the interventions used Ajzen’s theory of planned behavior or Ajzen and Fishbein’s theory of reasoned action in their theoretical basis. This is one of the main frameworks used in the design of evidence-based interventions [130]. It is considered one of the theories that allows the evaluation of adolescent sexual behavior [131]. This theory has shown that its two elements (intentions to have sex and perceived norms) are stable predictors of adolescent sexual behavior [132]. Likewise, studies have reported the effectiveness of interventions based on this theory in different kinds of sex-behavior-oriented interventions [133,134].

It should be noted that the second-most-used theoretical framework was Bandura’s social cognitive theory which, along with Ajzen’s theory, is a model that predicts and explains the mechanisms of behavior change, which is convenient in guiding the development of interventions [51]. Thus, it is possible to conclude that both social cognition models are crucial to successful A-SEIs.

In addition, it is important to highlight that 39% of the interventions in this review included two or more theories, which can also be an adequate way of designing an intervention. In order to resolve a problem through intervention, it is important to consider different aspects of the problem, and this is possible when using multiple theories [135].

Another core component is a participatory methodology, which was present in 94.4% of the A-SEIs. Some authors have pointed out that the most effective educational methods for teaching about sexuality are participatory and student-centered. This approach captures adolescents’ attention, leads to behavior change, and contributes to preparing them for life, favoring their health in a complex and changing world [72,73].

In terms of the type of intervention, most are targeted at mixed-sex groups (94.4%), which confirms the previous background that has shown that mixed-sex group interventions are more effective [38]. However, it must be noted that the review found one successful single-sex intervention.

The training of the facilitators was present in 94.4% of A-SEI. This component is one of the elements of the implementation’s support system that affects the implementation process [136]. It is critical to the success of educational interventions given that it enables the fidelity of implementation of the intervention’s essential elements [137]. When the facilitator follows the A-SEI model, favorable implementation outcomes are achieved [138]. Therefore, good results are produced when the training includes modeling and feedback in a supporting climate, as was seen in some of the interventions in this review [136].

The interventions varied regarding the dose. We were unable to establish standard criteria in connection with the number of sessions, but it is possible to point out that 39% of the A-SEIs delivered between 11 and 16 sessions. The total hours of intervention were, in general, similar as half of the interventions had between 10 and 19 h of exposure. These results are coherent with previous studies that highlight this variability [66].

The outcomes of the interventions included in this review were found to not only impact sex health, but also promote integral health, as they increased knowledge on different topics, promoted preventive sex behaviors, and increased favorable psychosocial outcomes.

None of the interventions that addressed gender reported specific outcomes linked to this topic. This prevented us from knowing if they impacted adolescents’ gender roles, gender relations, or gender identity. Including gender analysis is key to the interpretation and validation of research findings [139]. When there are no gender-related measures, it reduces the possibility of study replication and the effectiveness of research translation in health settings [140]. Some authors say the absence of gender-related measures is due to the scarcity of tools that evaluate how gender influences health outcomes [141] or due to the belief that the empirical measurement of gender is not clear [142].

### Limitations and Strengths

In terms of limitations, the review did not find articles with interventions implemented in Latin America or Asia with the quality criteria defined for this study. This could be related to the lack of resources to conduct RCTs in some developing countries. This restricts the knowledge of initiatives coming from these continents. However, it is important to mention that certain studies included in this review incorporated in their sample Hispanic/Latino and Asian participants [39,95,96,98,99,105,108,124]. Furthermore, regarding this limitation, it is relevant to stimulate studies about health topics such as SEI in these continents, especially in developing countries, as this is a priority for global health [143]. Even when this objective needs structural changes to be achieved [143], the dissemination of the review among Latin American researchers would contribute to enhancing the research capacity in this context.

Finally, the strengths of this review are: (1) the high quality of the studies included in this review and (2) the relatively high number of interventions analyzed.

## 5. Conclusions

During adolescence, SEIs are critical interventions as adolescents undergo physical and emotional changes, and sexuality becomes very important. Therefore, through SEIs, they can develop knowledge and skills to cope with sexual experiences to avoid the associated risks. In addition, as seen in this review, the outcomes of the A-SEIs are related to promoting preventive sexual behavior. Additionally, these interventions improve health in different areas (emotional, cognitive, and relational), which is crucial to adolescent development.

The results of this study identified the shared components of successful A-SEIs, which allows the suggestion of best practices in the design of and improvement in A-SEI. First, it is important to identify the behavior change theoretical models in the interventions, such as Ajzen’s theory of planned behavior, Ajzen and Fishbein’s theory of reasoned action, or Bandura’s social cognitive theory. These theories may contribute to increased protective sex behavior. However, in our opinion, it would be essential to include ecological approaches that address contextual and community elements, which are crucial determinants of sex behavior [144].

Second, as seen in the interventions reviewed, the use of the participatory methodology promotes the interaction, modeling, and developing of critical thinking. In this way, is an appropriate strategy for A-SEIs because adolescents need stimulating and reflexive activities to engage with the activities.

Third, interventions targeted at mixed-sex groups would be more appropriate in sex education for adolescents (although the intervention of Bangi [95] for female adolescents has favorable outcomes).

Fourth, the training of the facilitators must be included in A-SEIs. The relevance of this component is related to the implementation fidelity and skill development. When a facilitator knows the purpose and foundations of the intervention, they are more likely to be committed to carrying out the activities as they were designed. In addition, by having the skills to carry out an intervention in a participatory way, the facilitator will be more confident in performing it.

Finally, even though there is no standard dose in the SEI-A of this review, it can be recommended that at least ten hours of intervention should be delivered weekly. This component indicates that achieving favorable results in this kind of intervention requires a certain amount of time.

With respect to future research, the descriptive results of this review about the shared components of successful A-SEIs enable future meta-analysis to detect moderating factors more precisely.

## Figures and Tables

**Table 1:** Search Terms.

Main Terms	Search Terms
Sexuality	“sexuality” OR “sex” OR “HIV” OR “pregnancy” OR “STD” OR “abstinence” OR “reproductive” OR “sexually transmitted infection” OR “sexually transmitted disease” OR “STI” OR “AIDS” OR “reproductive health” OR ”condom” OR “contracept” OR “protected sex” OR “unprotected sex” OR ”abstinence” OR “safe sex”
Education	“education” OR “intervention” OR “program” OR “prevention” OR “treatment” OR “promotion”
Adolescent	“adolescent” OR “teenager” OR “teen” OR “juvenile” OR “youth” OR “young” OR “high school students” OR “middle school students” OR “girls” OR “boys”
Randomized control trial	“randomized control trial” OR “RCT” OR “randomized trial” OR “randomized clinical trial” OR “randomized controlled trial”

**Table 2:** Characteristics of included studies.

Intervention	Study	Jadad Scale Score	Sample Age (Years)	Setting	Country
“SAFETY” intervention	Jerlström et al., 2020 [91]	3	15	Educational	Sweden
Not specified	Angrist et al., 2019 [92]	3	12–14	Educational	Botswana (Kgatleng, Kweneng, South East, and Southern)
Comprehensive sexuality educational program	Yakubu et al., 2019 [93]	3	14–19	Educational	Ghana (Tamale Metropolis)
“Health Teacher” Family Health and Sexuality module	Goesling et al., 2016 [94]	3	12	Educational	United States (Chicago)
Project ÒRÉ	Bangi et al., 2013 [95]	3	14–18	Community-based	United States (San Francisco)
Reducing the Risk (RTR)	Barbee et al., 2016 [96]	5	14–19 years	Community-based	United States (Louisville, Kentucky)
Love Notes (LN)			14–19 years	Community-based	United States (Louisville, Kentucky)
Project PREPARED	Bauman et al., 2021 [97]	3	12–15 years	Clinical	United States (Bronx, New York)
High School FLASH 1 ^1^	Rohrbach et al., 2015 [98] Constantine et al., 2015 [39]	3	12–18 years	Educational	United States (Los Angeles)
High School FLASH 2 ^1^	Coyle et al., 2021 [99]	3	15 years	Educational	United States (Los Ángeles)
PREPARE 1 ^2^	Mathews et al., 2016 [100]	3	13 years (mean age)	Educational	South Africa (Western Cape)
PREPARE 2 ^2^	Mmbaga et al., 2017 [101]	3	12–14 years Tanzania	Educational	Tanzania (Dar es Salaam)
RTR +	Reyna and Mills, 2014 [102]	3	16 years (mean age)	Educational	United States (Arizona, Texas, and New York)
The HIV/STI risk-reduction intervention	Jemmott et al., 2016 [103]	3	12–18 years	Educational	South Africa (Eastern Cape Province)
Teen Outreach Program (TOP)	Walsh-Buhi et al., 2016 [104]	3	14–16 years	Educational	United States (Florida)
Comprehensive sexuality education intervention	Kemigisha et al., 2019 [26]	3	11–15 years	Educational	Uganda (Mbarara district)
Teenage Pregnancy PreventionProgram	Taylor et al., 2014 [105]	3	Males mean age: 14.6 years Females mean age: 13.9 years	Educational	South Africa (KwaZulu-Natal)
COMPAS (Competencias para adolescentes con una sexualidad saludable-Skills for Adolescents with a Healthy Sexuality)	Espada et al., 2012, 2015, 2017 [106,107,108]; Morales et al., 2015 [109]	3	15–18 years	Educational	Spain

NI = No information was found. ^1^ = Different randomized controlled trials (RCT). ^2^ = Same RCT in different countries.

**Table 3:** Sexuality education interventions’ characteristics.

Intervention	Number of Sessions and Frequency	Length of Session	Total Hours	Facilitators	Approach	Intervention Methodology	Type of Intervention	Facilitators Training
“SAFETY” intervention	One	80 min.	80 min.	Professional actors, staff from the municipality’s youth guidance center, and the school nurse	Risk-oriented	Participatory	Mixed-sex groups	Facilitators received training
Not specified [92]	Two	60 min.	60 min.	Youth facilitators	Risk-oriented	Participatory Video	Mixed-sex groups	5 days’ training
Comprehensive sexuality educational program	Six (two sessions weekly)	NI	NI	Qualified midwives	Comprehensive	Participatory	Mixed-sex groups	NI
“Health Teacher” Family Health and Sexuality module	Nine (the sessions were conducted for between two weeks and four months)	45–90 min.	10.1 h. (on average)	School teachers	Comprehensive	Participatory Video	Mixed-sex groups	3 days’ training
Project ÒRÉ	One	5 h.	5 h.	African American female health educators at community-basedorganizations	Comprehensive	Discussion Video	African American female adolescents	Facilitators received training
Reducing the Risk (RTR)	16 (weekly: the sessions were conducted on two days (two Saturdays))	Total hours distributed in two consecutive Saturdays	13 h.	Trained facilitators	Comprehensive	Participatory video	Mixed-sex groups	Facilitators received training
Love Notes (LN)	13 (weekly)		13 h.	Trained facilitators	Comprehensive	Participatory video	Mixed-sex groups	Facilitators received training
Project PREPARED	14 (weekly)	2 ¼ hours	35 h.	Trained facilitators	Comprehensive	Participatory	Mixed-sex groups	Facilitators received training
High School FLASH 1	12 (sessions were implemented across an average span of 53 days)	50 min.	10 h.	Planned Parenthood Los Angeles staff	Comprehensive	Participatory	Mixed-sex groups	2 days’ training
High School FLASH 2	15 (daily and alternate days)	50 min. 70–90 min.	12.9 h. (on average)	Sexuality educators from existing reproductive health and education organizations	Comprehensive	Participatory	Mixed-sex groups	2 days’ training
PREPARE 1	21 (weekly)	1–1.5 h	26.3 (on average)	Trained facilitators	Comprehensive	Participatory	Mixed-sex groups	2-week training course
PREPARE 2	25 (weekly)	40–80 min. 60–90 min.	19 h.	Teachers, peer educators, and healthcare providers	Comprehensive	Participatory–interactive	Mixed-sex groups	Facilitators received training
RTR +	15 (on average, a full intervention was implemented within 15.2 days)	2 h	16 h.	Research assistants	Abstinence-plus	Participatory–interactive	Mixed-sex groups	Over 16 h of training
The HIV/STI risk-reduction intervention	Six (daily)	1 h	12 h.	Women and men bilingual in English and Xhosa	Risk-oriented	Participatory–interactive	Mixed-sex groups	8 days’ training
Teen Outreach Program (TOP)	25 (weekly)	NI	NI	Trained teachers	Comprehensive	Participatory–interactive	Mixed-sex groups	Facilitators received training
Comprehensive sexuality education intervention	11 (montly)	1–2 h	16.5 h. (in avergae)	Volunteer university students	Comprehensive	Participatory–interactive	Mixed-sex groups	Facilitators received training
Teenage Pregnancy Prevention Program	12 (weekly)	1 h	24 (on average)	Trained facilitators	Comprehensive	Participatory–interactive video	Mixed-sex groups	Facilitators received training
COMPAS (Competencias para adolescentes con una sexualidad saludable-Skills for Adolescents with a Healthy Sexuality)	Five (weekly)	1 h	5 h.	Trained psychologists	Risk-oriented	Participatory–interactive	Mixed-sex groups	6 h training

**Table 4:** Theoretical frameworks of sexuality education interventions.

	Theory																
Intervention	Theory Of Planned Behavior [110]	Theory Of Reasoned Action [111,112]	Social Cognitive/ Learning Theory [113,114]	The Social Ecological Model [115]	Social Influence Theory [116]	Social Inoculation Theory [117]	Cognitive-Behavioral Theory	I-Change Model [118]	Empathy Model of Altruism [119]	Brecht’s Theory [120]	Health Belief Model	AIDS Risk Reduction Model [121]	Ecodevelopmental Theory [122,123]	Jewkes Conceptual Framework [124]	The Information-Motivation-Behavioral Skills Model [125]	Fuzzy-Trace Theory [126]	Positive Youth Development
**High School FLASH 1**	x			x													
**High School FLASH 2**	x																
**“SAFETY” intervention**										x							
**Not specified** [92]	NI																
**Comprehensive sexuality educational program**											x						
**“Health Teacher” Family Health and Sexuality module**			x				x										
**Project ÒRÉ**												x					
**RTR (Reducing the Risk)**	x		x		x	x	x		x								
**Love Notes (LN)**	x																
**Project PREPARED**	x	x	x										x				
**PREPARE programme 1**		x						x						x			
**PREPARE programme 2**		x	x					x						x			
**RTR +**	x		x		x	x	x		x							x	
**The HIV/STI risk-reduction intervention**	x		x														
**Teen Outreach Program (TOP)**																	x
**SRH intervention**	x			x													
**TP Program**								x									
**COMPAS**			x												x		

NI = No information was found.

**Table 5:** Interventions outcomes (statistically significant).

Name of the Intervention	Outcomes
**“SAFETY” intervention**	Increased knowledge on condom use, chlamydia, and protection. Better attitudes toward condoms. Less risky behavior about condom use.
**Not specified** [92]	Decreased pregnancy risk.
**Comprehensive sexuality educational program**	Increased knowledge on the use of contraceptives and susceptibility to adolescent pregnancy. Improved abstinence and intention to abstain from sex. Increased perceived severity of teenage pregnancy, perceived barriers to adolescent pregnancy prevention, perceived benefits of delaying pregnancy, and perceived self-efficacy.
**“Health Teacher” Family Health and Sexuality module**	Greater exposure to information on reproductive health topics. Higher knowledge of contraceptive methods and STI transmission.
**Project ÒRÉ**	Higher knowledge of HIV/STI prevention and protection. Greater knowledge of living with HIV/STI. Higher perceived HIV risk, perceived STI risk, and intentions to use condoms for vaginal sex.
**RTR** **(Reducing the Risk)**	Fewer sexual partners. Greater use of birth control.
**Love Notes** **(LN)**	Greater use of birth control and condoms. Fewer sexual partners. Less likelihood to have ever had sex.
**Project PREPARED**	Better HIV knowledge, sexual self-efficacy, and expectancy for condom use. Higher expectancy for condom use and intention for partner communication about HIV or STIs.
**High School FLASH 1**	Access to sexual health information. Awareness of sexual health services. More likelihood to have used sexual health services. More likelihood to be carrying a condom. Favorable attitudes about relationship rights. Higher levels of sexual health knowledge, self-efficacy to manage risky situations, and communication about sex.
**High School FLASH 2**	Greater knowledge about sexual health and sexual health services. More positive attitudes about sexual relationship rights. Greater communication about sex and relationships with parents. Greater self-efficacy to manage risky situations.
**PREPARE 1**	Better condom use and HIV knowledge. Lower rates of intimate partner violence.
**PREPARE 2**	Decreased incidence of sexual initiation. Higher condom use. Increased action planning for condom use.
**RTR +**	Lower intentions to have sex. Lower number of sexual partners. More intentions to use prophylaxis. Less favorable attitudes towards sex. Less permissive peer norms perceived. Knowledge about prophylaxis, sexual risk taking, pregnancy, and STIs. Greater self-efficacy for refusing sex and for prophylaxis (using contraception). Higher risk perception.
**The HIV/STI risk-reduction intervention**	Reduced self-reported unprotected sex. Increased self-reporting of talking to parents about not having sex.
**Teen Outreach Program (TOP)**	Lower odds of engaging in recent sex, risky sex, and intention to have sex.
**SRH intervention**	Improved sexual and reproductive health knowledge.
**Teenage Pregnancy Prevention Program**	Intentions to abstain from sex whilst at school. Plans to communicate with partners about teenage pregnancy. Increased condom use.
**COMPAS (Competencias para adolescentes con una sexualidad saludable-Skills for Adolescents with a Healthy Sexuality)**	More knowledge about HIV. Better attitudes toward condom use. More favorable attitudes toward HIV testing, and toward people living with HIV. Increased sexual risk perception. Increased perceptions about the peers’ consistent condom use. Age delay of the first time having intercourse.

## Data Availability

The following data are available upon request to the corresponding author: PRISMA checklist, template data collection forms and data extracted from included studies.

## Supplementary Materials

The following supporting information can be downloaded at: https://www.mdpi.com/article/10.3390/ijerph20054170/s1, File S1: Systematic Review Protocol; Table S2: Filters and limits used for the search.
