# Supplementary material for: Shared Components of Worldwide Successful Sexuality Education Interventions for Adolescents: A Systematic Review of Randomized Trials
Source: Int J Environ Res Public Health. 2023 Feb 25;20(5):4170. doi: 10.3390/ijerph20054170 (PMC10002383; doi:10.3390/ijerph20054170)
Supplement: Supplementary file 1 [file ijerph-20-04170-s001.zip › File S1.pdf]

Systematic Review Protocol

"Shared components of worldwide successful sexuality education interventions for adolescents: A systematic review of randomized trials"

Betzabé Torres-Cortés, Loreto Leiva, Katia Canenguez, Marcia Olhaberry and Emmanuel Méndez

Citation

Torres-Cortés, B., Leiva, L., Canenguez, K., Olhaberry, M. and Méndez, E. 2023. Shared components of worldwide successful sexuality education interventions for adolescents: A systematic review of randomized trials.

Review question

What are the shared components of effective sexuality education interventions for adolescents?

Searches

This systematic review was conducted using specific main keywords such as "Sexuality", "Education", "Adolescent", "Randomized control trial" between November and December 2021. We searched electronic databases CINAHL, PsycInfo, and PubMed databases; and the Web of Science [WoS] platform to find studies about sexuality education interventions for adolescents.

Types of study to be included

Randomized clinical trial

Condition or domain being studied

Sexuality education intervention: any combination of learning experiences aimed at developing a voluntary behavior leading to sexual health in adolescents. The intervention had to be universal, preventive, targeted to adolescents (11 to 19 years old), and only include sex health-related topics (from abstinence, risk-oriented or comprehensive approach).

Participants/population

We included any Randomized clinical trial about sexuality education interventions targeted to adolescents. This study only included interventions that targeted strictly sex behavior (and not those that addressed exclusively related topics as partner violence or sexual abuse).

Intervention(s), exposure(s)

Psychosocial intervention

Comparator(s)/control

Adolescents receiving sexuality education intervention and adolescents receiving other kind of health intervention

Main outcome(s)

Intervention components:

- Dose (number and length of sessions)
- Facilitator's training
- Theoretical foundations
- Intervention approach
- Type of intervention (individual or group; single-sex or mixed-sex groups)
- Activities methodology

Additional outcome(s)

Outcomes of the interventions

Data extraction (selection and coding)

Two authors -independently- screened all retrieved papers in different levels including title, abstract, and full text following PRISMA guidelines for reporting systematic reviews. We extracted following data:

- *Study variables:* author, year of publication, title, country of implementation, type of randomization, blinding, the existence of a control group, reasons for withdrawal, and sample characteristics (size, sex, gender, and age).
- *Intervention characteristics:* name of the intervention, country of implementation, objective, setting (communitarian, scholar, clinical), number and length of sessions, type of facilitators, facilitator's training, theoretical foundations, topics, type of intervention (individual or group; single-sex or mixed-sex groups), type of activities, and statistically significative outcomes.

Risk of bias (quality) assessment

- High-quality indicators were included in the eligibility criteria: experimental design and existence of control group.
- Jadad scale: is one of the most used for evaluating RCT. This scale contains items directly associated with the reduction of bias and classifies the study as high quality when this gets from three to five points [87].

Strategy for data synthesis

Structured approach: the interventions components of each study were coded in a databased on pre-specified criteria (e.g., approach: 1) abstinence, 2) comprehensive, 3) risk-oriented). In this way, those with similar codes were grouped into the same categories.

Analysis of subgroups or subsets

Non applicable

Contact details for further information

Betzabé Torres-Cortés

bctorres1@uc.cl

Organisational affiliation of the review

- Department of Psychology, Pontificia Universidad Católica de Chile, Av. Vicuña Mackenna 4860, Macul, Chile
- Department of Psychology, Faculty of Social Sciences, Universidad de Chile, Avenida Capitán Ignacio Carrera Pinto 1045, Ñuñoa, Chile

Review team members and their organisational affiliations

Betzabé Torres-Cortés[1,2,3], Loreto Leiva[2], Katia Canenguez[4,5], Marcia Olhaberry[1,3] and Emmanuel Méndez[1,6]

[1] Department of Psychology, Pontificia Universidad Católica de Chile, Av. Vicuña Mackenna 4860, Macul, Chile; bctorres1@uc.cl (B.T-C.); mpolhabe@uc.cl (M.O.)

[2] Department of Psychology, Faculty of Social Sciences, Universidad de Chile, Avenida Capitán Ignacio Carrera Pinto 1045, Ñuñoa, Chile; loretoleivab@u.uchile.cl

[3] Millennium Institute for Research on Depression and Personality (MIDAP), Av. Vicuña Mackenna 4860, Macul, Chile

[4] Department of Psychiatry, Massachusetts General Hospital, Yawkey 6A, 55 Fruit Street, Boston, MA, USA

[5] Department of Psychiatry, Harvard Medical School, address, Boston, MA, USA

[6] Department of Psychiatry, Faculty of Medicine, Universidad de Chile, Avenida La Paz 1003, Independencia; Chile

Type and method of review

Intervention, Systematic review

Anticipated or actual start date

November 2021

Anticipated completion date

January 2023

Funding sources/sponsors

The National Research and Development Agency (ANID)/ Scholarship Program / DOCTORADO BECAS CHILE / 2020-21202409

Health Research and Development National Fund (FONIS), Project SA21I0142

Conflicts of interest

The authors declare that the study was conducted in the absence of any commercial or financial relationship that would constitute a potential conflict of interest.

Language

English

Country

Chile

Subject index terms

Sexuality, Education, Adolescent, Randomized control trial
