# Supplementary material for: Shared Components of Worldwide Successful Sexuality Education Interventions for Adolescents: A Systematic Review of Randomized Trials
Source: Int J Environ Res Public Health. 2023 Feb 25;20(5):4170. doi: 10.3390/ijerph20054170 (PMC10002383; doi:10.3390/ijerph20054170)
Supplement: Supplementary file 1 [file ijerph-20-04170-s001.zip › Table S2.pdf]

TS2: Filters and limits used for the search

Database or platform	Filters and limits
Web of Science (WoS)	Selection of "All fields"<br>Set publication date (from 2011-01-01)
CINAHL	Deselect all search fields except from "Publication date"and "Age groups"<br>Set publication date (from January 2011)<br>Selection on "Age groups: Adolescence"
PsycInfo	Deselect all search fields except from "Publication date"and "Age groups"<br>Set publication date (from January 2011)<br>Set publication date (from January 2011)<br>Selection on "Age groups: Adolescence"
PubMed	Selection of "All fields"<br>Deselect all search fields<br>Select:<br>- Randomized Controlled Trial<br>- Custom range: from 2011-01-01<br>- Age: Adolescent (13-18 years)
